# Prognostic Factors After the First Recurrence of Ovarian Cancer

**DOI:** 10.3390/jcm14020470

**Published:** 2025-01-13

**Authors:** Patricia Salas Bolívar, Cristina Gonzalez-Benitez, María Carbonell López, Jesús Díez Sebastian, Alicia Hernández Gutiérrez, Ignacio Zapardiel

**Affiliations:** Gynecologic Oncology Unit, La Paz University Hospital, Paseo Castellana 261, 28046 Madrid, Spain; patricia.salasbolivar@hotmail.com (P.S.B.); crisgb2404@hotmail.com (C.G.-B.); mariacarbonell676@gmail.com (M.C.L.); jdiezs@salud.madrid.org (J.D.S.); aliciahernandezg@gmail.com (A.H.G.)

**Keywords:** ovarian cancer, prognostic factors, recurrence, survival

## Abstract

**Objective:** Ovarian cancer is the fifth most frequent tumor in women and the second most common gynecological cancer. Recurrence of ovarian cancer develops in up to 50–90% of patients within the first five years after diagnosis. Approximately 70% of patients with advanced disease will experience a relapse. The aim of this study was to assess prognostic factors that may predict a higher probability of additional recurrences, as well as their treatment and impact on disease-free and overall survival. **Method:** A retrospective observational study was conducted on patients diagnosed with recurrent ovarian cancer at the Gynecologic Oncology Unit of Hospital Universitario La Paz (Madrid, Spain) from January 2000 to December 2020. All variables related to the initial treatment, diagnosis, and management of the recurrence were collected and analyzed. **Results:** Data from 144 patients with recurrent ovarian cancer were analyzed. Statistically significant differences were found in disease-free survival between patients depending on initial tumor staging and primary treatment. Better outcomes were observed in patients with FIGO (International Federation of Gynecology and Obstetrics) stages I–II compared to those with FIGO stages III–IV at diagnosis (*p* = 0.021), as well as in patients who underwent primary cytoreduction compared to those who received neoadjuvant chemotherapy followed by interval surgery (*p* < 0.001). The disease-free interval was categorized into three periods, <6 months, 6–12 months, and ≥12 months, with greater survival observed in those with a longer disease-free interval (*p* < 0.001). After the first recurrence, two factors significantly influenced patient survival: the type of treatment received after the first recurrence (*p* < 0.001) and the type of chemotherapy regimen (*p* = 0.001). Complete cytoreduction during primary treatment was an independent prognostic factor and was related to better overall survival in patients where it was achieved (*p* = 0.004), regardless of the number of recurrences. **Conclusions:** The prognostic factors with impact on the survival of patients with a first recurrence of ovarian cancer are the following: treatment modality of the primary tumor, treatment modality of the recurrence, type of chemotherapy regimen, and disease-free interval from initial diagnosis to the first recurrence.

## 1. Introduction

Ovarian cancer is the fifth most frequent tumor in women [1] and the second most common gynecological cancer. Each year, approximately 200,000 women worldwide are diagnosed with ovarian cancer, leading to about 125,000 annual deaths [2]. The 5-year survival rate is around 30–40% [3].

The majority of patients are diagnosed at advanced stages of the disease (70% in stages III and IV FIGO) [4], when symptoms of peritoneal dissemination appear, such as pain, abdominal distension, ascites, detection of an abdominal mass, gastrointestinal symptoms, and constitutional syndrome [5], resulting in lower patient survival. The 5-year survival in advanced stages with metastatic disease is around 25% [6]. Treatment of advanced-stage ovarian cancer is based on surgery aiming for complete disease resection, followed by platinum-based chemotherapy and, in certain cases, subsequent immunotherapy [7]. Residual disease after surgery is directly related to the risk of recurrence and overall survival of patients [8]. Tumor recurrences occur in up to 70% of patients with advanced disease during the first five years of follow-up [9], rendering their management crucial for patient survival.

The pattern of recurrence is highly variable, ranging from isolated foci of peritoneal disease, exclusive nodal involvement, and peritoneal carcinomatosis, to less frequent extra-abdominal foci. Therefore, the treatment of ovarian cancer recurrences is also highly variable. The main goals of care include controlling disease-related symptoms, maintaining or improving patient quality of life, increasing time to progression, and improving survival. Treatment depends on the extent and location of the recurrence. Surgery (complete cytoreduction) alone or in combination with platinum-based chemotherapy remain the principal therapeutic options. The platinum-free interval (PFI) has been the principal indicator for classifying tumors as “treatment-sensitive” or “resistant,” based on a 6-month cutoff from the last platinum-based therapy, supporting its use as the first medical treatment option [10].

This study was designed to evaluate the presence of prognostic factors in patients with recurrent ovarian cancer that may predict a higher probability of additional recurrences, to assess their therapeutic management, and to determine their impact on disease-free and overall survival. The aim of this study was to evaluate prognostic factors that could predict the probability of recurrence in ovarian cancer.

## 2. Materials and Methods

A retrospective observational study was conducted on patients diagnosed with recurrent ovarian cancer in the Gynecologic Oncology Unit of Hospital Universitario La Paz (Madrid, Spain) from January 2000 to December 2020. This study was reviewed and approved by the institutional ethics committee (reference number PI-4721).

All patients diagnosed with at least one recurrence of ovarian cancer were included in this study, provided that both the initial diagnosis and treatment occurred within our center. Patients diagnosed at our center who were lost to follow-up during this study were excluded.

All cases were presented at the Multidisciplinary Gynecological Tumor Board to discuss the optimal therapeutic approach for each patient among the different specialty teams involved, including gynecology, medical oncology, radiation oncology, pathology, radiology, and surgery. Clinical staging of the disease was performed using the 2014 International Federation of Gynecology and Obstetrics (FIGO) classification [11].

For all patients scheduled for surgery of the recurrence, a preoperative study was conducted, including physical examination and blood analysis (including tumor markers CA 125, CA 19.9, and CEA), pelvic and abdominal ultrasound, and thoracic abdominopelvic CT scan and/or PET-CT. The surgical treatment aimed to achieve complete tumor resection. The cytoreductive nomenclature used followed the criteria described by Zapardiel and Morrow (2011) [12], defining optimal cytoreduction as the reduction of tumor burden to nodules measuring less than 1 cm in maximum diameter. Relative contraindications for surgery included an ASA (American Society of Anesthesiologists) score >4, extensive infiltration of the root of the small bowel mesentery, extensive implants on the serosa of the small bowel, nodal involvement of the celiac trunk, unresectable involvement of the hepatic portal vein, multiple extra-abdominal metastases, or a platinum-free interval of less than 6 months. Secondary cytoreduction was also excluded for patients presenting extensive carcinomatosis at the time of recurrence and a disease-free interval of less than 12 months.

Patients for whom cytoreductive surgery was not possible received chemotherapy. In selected and exceptional cases, surgical reassessment was considered after three cycles of cisplatin/carboplatin–taxol before continuing medical treatment.

Disease-free survival was defined as the time in months from the treatment of the recurrence, defined as the date of surgery or the end of chemotherapy, until the appearance of a new recurrence. Overall survival was defined as the time in months from the treatment of the recurrence, defined as the date of surgery or the end of chemotherapy, until death or last contact with the patient.

After treatment, patients were followed up in the Gynecologic Oncology clinic every 6 months for early detection of recurrences with thorough gynecological examination, blood analysis including tumor markers, and a thoracic abdominopelvic CT scan.

An analysis of risk factors for subsequent recurrences was conducted, including those related to the primary tumor, histopathological characteristics, disease stage at diagnosis, initial treatment modalities received, and the baseline characteristics of the patients. Additionally, treatments for subsequent recurrences were included, specifically assessing the type of chemotherapy or surgery administered following the diagnosis of the initial recurrence. In essence, this analysis aimed to evaluate the risk factors influencing the likelihood of recurrence, encompassing factors related to the primary tumor as well as treatments for recurrences (surgery or type of chemotherapy).

### Statistical Analysis

Quantitative data were presented as means and standard deviations, and qualitative variables as absolute values and percentages. Comparisons between groups of qualitative variables were performed using the Chi-square test or Fisher’s exact test, and quantitative variables with the Student’s *t*-test or ANOVA. For multivariate analysis, a logistic regression model was used. Survival analysis was performed using the Kaplan–Meier method and log-rank tests. All comparisons were two-sided, and the alpha error was set at 5%. Statistical analysis was conducted using SPSS software v.23 (IBM Corp, Armonk, NY, USA).

## 3. Results

A total of 140 patients with recurrent ovarian cancer were included in this study. Baseline characteristics are shown in Table 1. The mean age of subjects was 57.9 ± 13.3 years. The histological subtypes included 106 (75.7%) serous, 13 (9.3%) endometrioid, 11 (7.9%) clear cell, and 7 (5%) mucinous tumors. The FIGO stage at initial diagnosis was stage I in 17 patients (12.1%), stage II in 5 (3.5%), stage III in 89 (63.5%), and stage IV in 18 (12.5%). In our series, most patients (76%) were diagnosed at advanced stages (stages III and IV).

Regarding the treatment for the primary tumor (prior to the first recurrence), 90 patients (64.3%) underwent cytoreductive surgery and adjuvant chemotherapy, 39 (27.9%) received neoadjuvant chemotherapy and interval surgery, 9 (6.4%) had surgery alone, and 2 (1.4%) received only chemotherapy. Of the 9 patients who did not receive subsequent chemotherapy, 3 were early-stage cases (2 stage IA and 1 stage IB) who underwent staging surgery, 2 were stage IIIC patients who underwent only primary cytoreductive surgery due to contraindications for adjuvant chemotherapy, and 4 were stage IA and IC patients who underwent only adnexectomy for fertility preservation.

Of the 140 patients included, 41 had 1 recurrence (29.2%), 35 (25%) had 2 recurrences, 27 patients (19.2%) had 3 recurrences, and 37 patients (26.4%) had 4 or more recurrences. Recurrences were classified as local in 82 patients (58.57%), nodal in 67 patients (47.8%), and distant in 59 patients (42.1%).

A multivariate analysis was performed on all factors collected that could be related to survival, as shown in Table 2.

**Figure 1:**
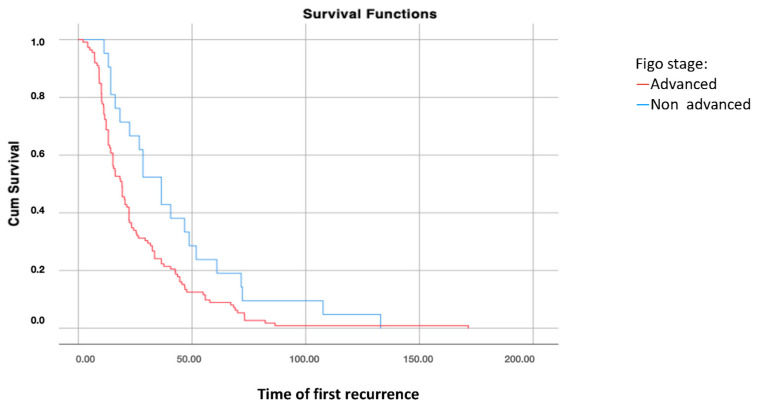
Disease-free survival 2: analysis using Kaplan–Meier curve according to the FIGO stage of the disease (log-rank: *p* = 0.021).

Survival analysis using the Kaplan–Meier curve according to FIGO stages at diagnosis showed significant differences in disease-free survival after the first recurrence (DFS2) between patients with FIGO stages I–II and those with FIGO stages III–IV at diagnosis (*p* = 0.021), with higher survival rates in patients with early-stage disease (Figure 1).

However, no significant differences were found in overall survival according to the FIGO stages at diagnosis (*p* = 0.581).

Differences were also found in disease-free survival (DFS1) between patients where complete cytoreduction was achieved versus those where it was not possible (*p* = 0.004). Of the 99 patients who underwent primary cytoreductive surgery, complete surgical eradication was achieved in 72 (72.7% complete cytoreduction rate).

**Figure 2:**
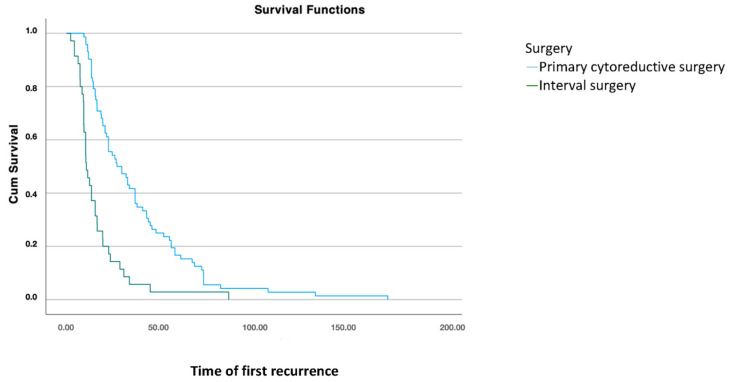
Disease-free survival 2: analysis using Kaplan–Meier curve according to treatment modality for the primary tumor (log-rank: 0.001).

Once the patient has recurred, comparing different treatments at initial diagnosis, disease-free survival (DFS2) was significantly higher in patients who underwent cytoreductive surgery compared to those who had interval surgery after neoadjuvant chemotherapy (*p* < 0.001) (Figure 2).

The mean disease-free survival from surgery to the diagnosis of the first recurrence was 28.9 ± 25.3 months. The median overall survival of the patients was 85.2 ± 53.4 months, with a 5-year survival rate of 57.7%.

**Figure 3:**
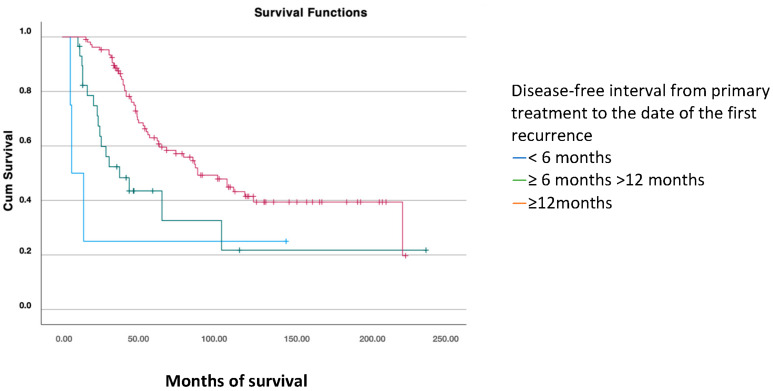
Overall survival analysis using the Kaplan–Meier curve according to the disease-free interval (PFS1) (log-rank: *p* < 0.001).

The disease-free interval (DFS1) from primary treatment to the date of the first recurrence was classified into three categories: <6 months, between 6 and 12 months, and ≥12 months. Significant differences were found, with greater overall survival in patients where the first recurrence occurred more than 12 months after primary treatment (mean of 124 months) versus 42 months in patients with a recurrence within the first six months and 82 months in those who recurred between 6 and 12 months (*p* < 0.001) (Figure 3).

**Figure 4:**
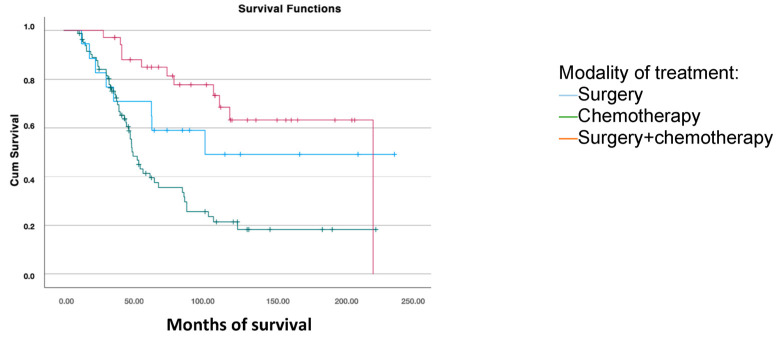
Disease-free survival analysis using the Kaplan–Meier curve according to the treatment modality for the first recurrence (log-rank: *p* < 0.001).

For survival analysis based on the treatment received after the first recurrence, patients were grouped into those who received chemotherapy, combined surgery and chemotherapy, and those who underwent surgical treatment alone. Significant differences were found, with greater overall survival in patients who underwent surgery combined with subsequent chemotherapy (168.6 months) compared to those treated only with surgery (141.2 months) or chemotherapy (83.1 months) (*p* < 0.001) (Figure 4).

Regarding the chemotherapy regimen received after recurrence, patients were categorized as having received carboplatin–paclitaxel versus other chemotherapy types (paclitaxel alone, carboplatin alone, carboplatin–gemcitabine, doxorubicin, or carboplatin–doxorubicin). Significant differences were found, with greater survival in patients who received carboplatin–paclitaxel (*p* = 0.001), with a mean survival of 148.7 months versus 76.5 months in those who received other chemotherapy types.

## 4. Discussion

A significant proportion of patients with ovarian cancer will suffer a recurrence (50–90% at 5 years from initial diagnosis). However, it remains a challenge to identify which factors can impact survival after the recurrence. The aim of this study was to identify and analyze these factors to improve the management of patients with recurrent ovarian cancer.

In our study, we analyzed the clinical characteristics and overall and disease-free survival in patients with recurrent ovarian cancer.

Factors like age, histological type, concurrent medical conditions, tumor size or tumor grade do not have an influence on the prognosis at recurrence. Regarding the factors that do impact survival, these may have a different impact on disease free survival (DFS2) or on overall survival (OS).

### 4.1. DFS2

In our study, the majority of patients (90%) underwent surgery and chemotherapy as primary treatment, with 64.3% receiving primary cytoreductive surgery and 27.9% undergoing interval surgery after neoadjuvant chemotherapy. Ten patients received surgery alone due to early-stage diagnosis, consistent with literature reports. Melamed et al. [13] reported similar percentages, with 66.4% undergoing primary surgery and 13.3% receiving neoadjuvant chemotherapy followed by interval surgery.

Most of our patients were diagnosed and treated after the publication of the 2010 Vergote study on neoadjuvant chemotherapy [14] and subsequent studies [8] that showed equivalent survival between patients undergoing primary cytoreduction and those having interval surgery, provided optimal surgery with macroscopic resection was achieved in both [8,13]. However, in our series, greater overall survival was found in patients who underwent optimal primary cytoreductive surgery (127 months) compared to those who received neoadjuvant chemotherapy (86 months) and subsequent optimal surgery. Differences in survival may be due to the allocation of patients with poorer prognosis—those with greater peritoneal involvement and biological aggressiveness—who were assigned to the group receiving prior chemotherapy and subsequent interval surgery. Additionally, the extensive experience of our surgical team in achieving maximal effort cytoreductive surgery at initial diagnosis may have improved patient survival, a hypothesis shared by other authors like Furuya et al. (2012) [15].

In our series, complete cytoreduction at initial diagnosis was achieved in 74% of patients, with disease-free survival of up to 36 months in these patients versus 22 months in those where complete cytoreduction was not possible. Bristow et al. [16] analyzed 81 studies involving 6885 ovarian cancer patients, establishing a mean survival of 33.9 months in cohorts with up to 75% complete cytoreduction rates. Complete cytoreduction remains one of the most important prognostic factors and is the primary objective of all surgical procedures [17].

In our study we have found that treatment at the time of initial diagnosis is one of the factors that impacts DFS2. Patients who underwent surgery at diagnosis have a better disease free survival after the first recurrence (DFS2) than those who received interval surgery (26.8 months vs. 10.5 months).

In our series of 140 patients, the majority were diagnosed at an advanced stage (77.7%), consistent with data found in other studies [18,19]. Typically, 70% of patients are diagnosed at advanced stages [20,21], with median overall survival ranging from 15 to 23 months for FIGO stage IV patients [22]. In our series, the median overall survival was 106 months for early stages and 126 months for advanced stages, with no statistically significant differences between the two groups. Given that all our patients had at least one recurrence, our data may suggest that, although prognosis at initial diagnosis is associated with stage, overall survival after the first recurrence is similar regardless of initial staging.

However, initial tumor staging does influence disease free survival after the first recurrence (DFS2); we did find statistically significant differences in DFS2 between early and advanced stages in our series, with means of 42 months for early stages and 26 months for advanced stages.

### 4.2. OS

The overall survival of our patients averaged 49 months, with a 5-year survival rate of 57.7%; median survival was 85.2 months and mean survival was 116.79 months. The overall survival in our series is slightly higher than that reported in other studies, which document 5-year survival rates of 49.7% [19] or 46.1% [23]. These differences may be due to patient selection bias in these studies; for instance, Dinca et al. included only patients in FIGO stages III and IV, resulting in a slightly lower 5-year overall survival rate.

After the first recurrence, greater overall survival was observed in patients whose first recurrence occurred more than 12 months after primary treatment (*p* < 0.001). For these patients, the median overall survival was 124 months, compared to 42 months for those with a recurrence within 6 months, and 80 months for those who recurred between 6 and 12 months. Therefore, the longer the progression-free interval after primary treatment—including platinum-based chemotherapy—the greater the likelihood of survival and response to second line chemotherapy. Similar findings are reported in the literature, such as in the study published by Eisenkop et al., showing a median survival of 25 months for recurrences between 6 and 12 months, 44 months for 13–36 months, and 56 months for beyond 36 months [24].

We also evaluated survival based on the treatment received after the first recurrence, differentiating between patients who received surgical treatment alone, those who received combined surgery and chemotherapy, and those who received chemotherapy alone. The median overall survival was 83 months for patients treated with chemotherapy, 141 months for those treated only with surgery, and 168 months for those who received both surgery and chemotherapy. Previous studies by Eisenkop et al. (2000) and Chi et al. (2006) [24,25] correlate an increased survival to achieving optimal cytoreduction during surgery of the recurrence, independently of other treatments received. However, the randomized DESKTOP-III trial [26] demonstrated an overall survival advantage in patients undergoing secondary cytoreductive surgery followed by chemotherapy, compared to chemotherapy alone, particularly when optimal cytoreduction was achieved. A limitation of this study is it included only patients who had cytoreductive surgery at initial diagnosis, excluding those who had neoadjuvant chemotherapy followed by interval surgery. A 2022 study analyzing survival in 272 patients undergoing secondary surgery for recurrence concluded that there are no statistically significant differences dependent on the primary treatment received (primary cytoreductive surgery vs. neoadjuvant chemotherapy and subsequent interval surgery), with a median survival of 21 months in both groups (*p* = 0.648) [27]. These findings may be explained by the response to chemotherapy during primary treatment, with patients who responded well to the initial platinum-based chemotherapy also responding better upon recurrence, thus being platinum-sensitive and exhibiting greater overall survival compared to those with platinum resistance who received alternative chemotherapy regimens.

In our study, significant differences in overall survival were also found based on the type of chemotherapy administered after the first recurrence, distinguishing between patients who received carboplatin–paclitaxel and those who received other chemotherapy regimens. Patients treated with carboplatin–paclitaxel had significantly greater overall survival (148 months vs. 76 months; *p* = 0.001). Similarly, Colombo et al. [22] also reported greater overall survival for patients treated with platinum-based chemotherapy regimens (OS [hazard ratio (HR) 0.71; 95% confidence interval (CI) 0.53–0.93] and PFS (HR 0.67; 95% CI 0.53–0.84]). Two large randomized multicenter studies, OV-10 and GOG 111, demonstrated the superiority of the cisplatin–paclitaxel regimen over previous regimens in terms of progression-free and overall survival, findings later confirmed with the addition of bevacizumab or PARP inhibitors in various studies [28,29,30]. Burger et al. [29] observed an increase in progression-free survival of up to 4 months in patients treated with carboplatin–paclitaxel plus bevacizumab, and Perren et al. reported an increase in median survival from 28.8 months without bevacizumab to 36.3 months with its addition [30]. Cheng et al. (2017) established cisplatin–paclitaxel as a safe and effective treatment for advanced ovarian cancer, with mean and median progression-free survival rates of 36.5 and 27.0 months, respectively (95% CI: 26.8–46.2 and 11.3–42.7 months) [31]. Vergote et al. (2022), in the 6th Ovarian Cancer Consensus of the Gynecologic Cancer InterGroup (GCIG), recommended platinum-based regimens as the first treatment option for patients who responded to these chemotherapy regimens initially [23].

## 5. Conclusions

Among the prognostic factors analyzed, the type of treatment and FIGO stage at initial diagnosis were found to significantly impact disease-free survival after the first recurrence (DFS2). Primary cytoreduction demonstrated superior outcomes compared to interval debulking surgery, as did early-stage disease.

In contrast, overall survival (OS) was not influenced by the FIGO stage at diagnosis, suggesting that once a patient experiences a recurrence, OS becomes independent of the initial stage. Factors associated with improved OS included recurrence interval greater than 12 months, the use of surgical treatment followed by chemotherapy for the recurrence, and the administration of carboplatin–paclitaxel.

## Figures and Tables

**Table 1:** Baseline characteristics of patients with recurrent ovarian cancer at initial diagnosis (*n* = 140).

Age (yr)	Median (Range)	57.96 ± 13.3 years
Epidemiological factors	Parity: Nulliparous/Multiparous	47 (32.6%)/97 (67.3%)
	Menopause: Yes/No	104 (74.3%)/36 (25.7%)
	HTN: Yes/No	36 (25.7%)/104 (74.3%)
	Diabetes mellitus: Yes/No	11 (7.9%)/129 (92.1%)
	Breast cancer: Yes/No	6 (4.3%)/134 (95.7%)
	BRCA: 1/2	2 (1.4%)/4 (2.9%)
	Obesity: Yes/No	1 (0.7%)/139 (99.3%)
FIGO stage	I	18 (12.9%)
	II	5 (3.5%)
	III	89 (63.5%)
	IV	18 (12.9%)
	Missing	10 (7.1%)
Grading of tumor	Low grade	33 (22.9%)
	High grade	96 (68.6%)
	Non grade	11 (7.9%)
Size of tumor	≥10 cm	68 (48.6%)
	≤10 cm	45 (32.1%)
	Unknown	27 (19.3%)
Histological type	Epithelial ovarian cancer - Serous - Endometrioid - Clear cell - Mucinous - Unknown	106 (75.7%) 13 (9%) 11 (7.6%) 7 (4.9%) 3 (2.1%)
Treatment at initial diagnosis	Surgery + adjuvant chemotherapy	90 (64.3%)
	Neoadjuvant chemotherapy + interval surgery	39 (27.9%)
	Surgery	9 (6.4%)
	Chemotherapy	2(1.4%)
Type of chemotherapy at initial diagnosis	Yes	126 (90%)
	Missing	3 (2.1%)
	Paclitaxel + carboplatin	105 (75%)
	Taxol	4 (2.9%)
	Carboplatin	6 (4.3%)
	Carboplatin + tamoxifen	1 (0.7%)
	Carboplatin + doxorubicin	2 (1.4%)
	Bevacizumab + carboplatin + taxol	8 (5.7%)
	Others	3 (2.1%)
Treatment of the first recurrence	Surgery	18 (12.9%)
	Chemotherapy	83 (59.3%)
	Surgery + chemotherapy	35 (25%)
	Palliative treatment	2 (1.4%)
	Radiotherapy	1 (0.7%)
	No treatment	1 (0.7%)
Type of chemotherapy of first recurrence	Yes	117 (83.6%)
	Missing	1
	Paclitaxel + carboplatin	36 (25.7%)
	Taxol	4 (2.1%)
	Carboplatin	7 (5.0%)
	Carboplatin + gemcitabine	21 (15%)
	Doxorubicin	14 (11.5%)
	Carboplatin + doxorubicin	18 (12.9%)
	Bevacizumab	9 (6.4%)
	Others	8 (5.7%)
Optimal cytoreduction of the first recurrence		33 (62.2%)
Other recurrences	Second recurrence	101 (72.1%)
	Third recurrence	65 (64.3%)
	Fourth recurrence	37 (56.9%)

**Table 2:** Prognostic factors.

Epidemiological Data	N		Survival (Months)		*p* Value
Age	<50 yr	26.3%			0.027
	50–59 yr	22.9%			
	60–69 yr	27.0%			
	≥70 yr	22.9%			
Parity	Yes	32.63%			0.501
	No	67.36%			
Menopause	Yes	25.7%			0.375
	No	74.3%			
HTA	Yes	25.7%			0.169
	No	74.3%			
Diabetes	Yes	7.9%			0.988
	No	92.1%			
Breast cancer	Yes	4.3%			0.613
	No	95.7%			
BRCA 1/2	Yes	4.3%			0.521
	No	95.7%			
Obesity	Yes	0.7%			0.820
	No	99.3%			
Histological type	Epithelial				0.046
	Serous	(75.7%)			
	Endometrioid	13 (9%)			
	Clear cell	11 (7.6%)			
	Mucinous	7 (4.9%)			
	Unknown	3 (2.1%)			
Size	<10 cm	32.1%			0.972
	≥10 cm	48.6%			
Grading of tumor	Low	22.9%			0.840
	High	68.6%			
	No grade	7.9%			
Treatment at initial diagnosis	Primary cytoreductive surgery	64%	DFS2	26.8 m	0.000
	Interval surgery	28%	DFS2	10.5 m	
	Others	8%			
Treatment after the first recurrence	Surgery	12.9%	OS	141.2 m	0.000
	Chemotherapy	59.3%	OS	83.1 m	
	Surgery + chemotherapy	25%	OS	168.6 m	
Type of chemotherapy—first therapy	Carboplatin–taxol	31.07%	OS	148.7 m	0.001
	Others	68.93 %	OS	76.5 m	
Disease free survival (DFS)	<6 m	2.8%	OS	42.7 m	0.001
	≥6–<12 m	20.7%	OS	82.8 m	
	≥12 m	76.4%	OS	124.4 m	
Stage FIGO (DFS2)	Early	16.6%	DFS2	36.5 m	0.0021
	Advanced	77.7%	DFS2	19.2 m	
	Unknown	5.6%			
FIGO Stage (OS)	Early	16%			0.581
	Advanced	77.7%			
	Unknown	5.6%			

## Data Availability

The original contributions presented in the study are included in the article, further inquiries can be directed to the corresponding author.
